# Prospection of plant-based bio-insecticides for mosquito vector control in Tanzania: A comprehensive review

**DOI:** 10.1016/j.parepi.2026.e00505

**Published:** 2026-04-17

**Authors:** Deokary Joseph Matiya

**Affiliations:** Dar es Salaam University College of Education (DUCE), University of Dar Es Salaam, P.O. Box 2329, Dar es Salaam, Tanzania

**Keywords:** Plant bio-insecticides, Mosquito control, Plant extracts, Larvicidal activity, Tanzanian flora

## Abstract

Mosquito-borne diseases, including malaria, dengue, chikungunya, zika, and lymphatic filariasis, pose significant public health challenges in Tanzania. Current vector control strategies largely depend on synthetic insecticides, but the emergence of insecticide resistance, environmental persistence, and non-target toxicity necessitates alternative, eco-friendly solutions. Plant-based bioinsecticides are gaining attention due to their biodegradability, low mammalian toxicity, and complex phytochemical profiles that hinder resistance development. This systematic review synthesizes 41 studies conducted in Tanzania up to November 2025, evaluating indigenous plant species for insecticidal and repellency activities against mosquito vectors. A total of 74 plant species from 22 families were identified across 13 Tanzanian regions, with 46 assessed for insecticidal activity. Leaves were the most utilized plant part, while sequential solvent extraction and essential oil isolation via steam distillation and hydro-distillation were the predominant extraction techniques. Larvicidal assays dominated, with 58% of tested extracts exhibiting high potency (LC₅₀ < 100 μg/mL). Plant species with high larvicidal activity included *Commiphora merkeri*, *Dioscorea sansibarensis*, *Tephrosia vogelii*, *Uvariodendron pycnophyllum*, *Harrisonia abyssinica*, *Annona squamosa*, *Annona senegalensis*, *Annona muricata*, and *Neorautanenia mitis*. Highly promising species for adulticidal activity and repellency were *Warburgia ugandensis*, *Neorautanenia mitis*, *Tessmannia densiflora*, *Chenopodium ambrosioides*, *Ocimum* spp., *Syzygium aromaticum*, and *Cinnamomum verum*. Despite encouraging findings, most studies remain laboratory-based, emphasizing the need for expanded field trials, improved extraction methods, and formulation development to harness Tanzania's rich plant biodiversity for sustainable mosquito vector control.

## 1 Introduction

Mosquito-borne diseases remain a significant global health concern. *Anopheles* mosquitoes transmit malaria, with an estimated 263 million malaria cases annually (WHO, 2024a). *Aedes* mosquitoes are linked to about 96 million dengue, 693,000 chikungunya, 500,000 Zika, and 130,000 yellow fever cases, while *Culex* mosquitoes are responsible for about 38.5 million lymphatic filariasis, 42,500 Japanese encephalitis, and 2588 West Nile fever cases (WHO, 2017). In Tanzania, 8.6 million malaria cases were reported in 2023 (WHO, 2024a), alongside 6917 dengue cases in 2019 and high seroprevalence of chikungunya (28%) and Zika (6.8%) (Mwanyika et al., 2021a; Mwanyika et al., 2021b). Lymphatic filariasis affects approximately 6 million people in the country (Fimbo et al., 2020). These figures highlight the urgent need for effective and sustained control measures against mosquito-borne diseases in the country.

Mosquito-borne disease control primarily relies on synthetic insecticides targeting adult mosquitoes, with the two main strategies being Long Lasting Insecticidal Nets (LLINs) and Indoor Residual Spraying (IRS). LLINs mostly use pyrethroids, although newer versions combine two insecticides for improved effectiveness (WHO, 2024a). IRS employs a broader range, using insecticides from six classes: pyrethroids, organophosphates, organochlorines, carbamates, neonicotinoids, and broflanilide (WHO, 2024b). In addition to large-scale strategies, supplementary interventions include the use of synthetic repellents, particularly N, N-diethyl-meta-toluamide (DEET), applied topically to prevent mosquito bites during outdoor exposure or in areas beyond the reach of LLINs and IRS. Larval control measures are also employed, using either synthetic insecticides or biolarvicides, especially *Bacillus thuringiensis* var. *israelensis* (Bti) and *Bacillus sphaericus* (Bs) (Gavana et al., 2025). These strategies have significantly contributed to the reduction of mosquito-borne diseases in various endemic regions (Musoke et al., 2023).

However, the widespread and prolonged use of a limited number of insecticide classes has led to the rapid emergence of resistance among mosquito populations, undermining the effectiveness of current control efforts (Matiya et al., 2019; van den Berg et al., 2021). Furthermore, many of these synthetic chemicals pose risks to human health and the environment, adversely affecting terrestrial and aquatic ecosystems, including non-target organisms, natural enemies, and decomposers, due to their high toxicity and environmental persistence (Zhou et al., 2025). Therefore, there is an urgent need to develop and implement alternative vector control products that are both effective and environmentally sustainable.

Plant-derived biopesticides are increasingly recognized as sustainable tools for mosquito and insect vector control (Hillary et al., 2024). Their inherent role in plant defense, low toxicity to humans, mammals, and non-target invertebrates, along with their biodegradability, host specificity, and minimal environmental impact, make them well-suited for Integrated Vector Management (IVM) (Khursheed et al., 2022). Their multicomponent nature enhances efficacy and makes it more difficult for insects to develop resistance compared to single-compound synthetic pesticides (Pavela et al., 2019). Although interest declined following the rise of synthetic pesticides in the mid-20th century, growing concerns about environmental and health risks have renewed focus on safe and effective plant-based alternatives (Senthil-Nathan, 2020).

Several plant species have been investigated and utilized in the development of commercial botanical pesticides in agriculture, including pyrethrum daisy (*Tanacetum cinerariifolium*), tobacco (*Nicotiana tabacum*), garlic (*Allium sativum*), neem (*Azadirachta indica*), chilli pepper (*Capsicum annuum*), sweetsop (*Annona squamosa*), Chinese bittersweet (*Celastrus angulatus*), sweet orange (*Citrus sinensis*), fish poison vine (*Derris elliptica*), pongam tree (*Pongamia pinnata*), sabadilla (*Schoenocaulon officinale*), thyme (*Thymus vulgaris*), and oregano (*Origanum vulgare*) (Chaudhary et al., 2024). Similarly, in vector control, repellent products have been developed from the essential oils of lemon grass (*Cymbopogon* spp.) and lemon eucalyptus (*Corymbia citriodora*) (Maia and Moore, 2011), highlighting the potential of plant-derived biopesticides in mosquito-borne disease interventions.

Building on these advances, ongoing research across the globe continues to screen plant extracts and their constituents for insecticidal activity against mosquito vectors (Pavela et al., 2019; Asadollahi et al., 2019). These investigations evaluate a range of bioactivities, including ovicidal, larvicidal, pupicidal, and adulticidal effects, as well as sublethal impacts such as antifeedant activity, growth regulation, and effects on fecundity and development (Hillary et al., 2024; Pavela et al., 2019; Asadollahi et al., 2019; Pavela, 2015; Collares et al., 2023). However, the potency of these extracts can vary considerably even within the same plant species, influenced by multiple factors such as chemo-types, genetic variation, plant variety, environmental conditions, geographical origin, plant part used, stage of maturity, harvest time, and extraction method (Innocent and Magadula, 2010; Daniel et al., 2011; Benomari et al., 2023). Therefore, it is essential to screen plant materials from diverse ecological and geographical backgrounds to identify the most bioactive chemo-types for effective vector control strategies.

In Tanzania, research on plant-based bio-insecticides has gained momentum since the early 2000s, with increasing efforts directed toward evaluating their efficacy against mosquito vectors. Numerous studies have investigated the insecticidal potential of indigenous and locally available plant species, demonstrating their promise as sustainable alternatives to synthetic insecticides. These studies have explored a range of bioactivities, including larvicidal, repellency and adulticidal effects. However, the existing body of literature remains fragmented, with considerable variation in the plant species assessed, the extraction methods employed, the mosquito life stages targeted, and the bioassay protocols used. This lack of synthesis hinders the identification of consistently effective plant species and constrains the development of evidence-based plant-derived bio-insecticide products for mosquito control.

A systematic review is therefore appropriate to bring together existing knowledge, highlight research patterns, and point out areas that may benefit from further study. This review can help provide a valuable overview to support future research efforts, inform public health discussions, and contribute to the exploration of plant-based vector control options in Tanzania and more widely in bordering countries in east Africa. Accordingly, this study aims to review and summarize the available literature on plant-based bio-insecticides evaluated against mosquito vectors in Tanzania, to outline observed patterns, identify species of interest, and suggest possible directions for further research.

## 2 Material and methods

### 2.1 Literature search

**Fig. 1:**
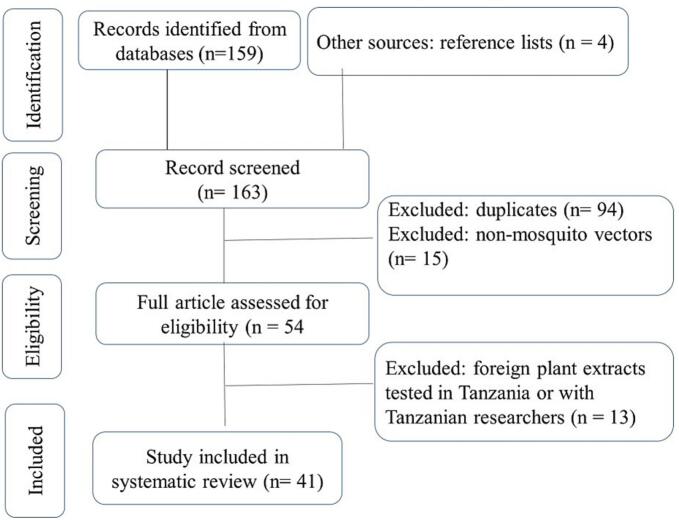
Flowchart of the article selection process based on PRISMA guidelines.

The literature search was conducted in accordance with the Preferred Reporting Items for Systematic Reviews and Meta-Analyses (PRISMA) guidelines, as illustrated in Fig. 1. Relevant peer-reviewed articles published up to November 2025 were systematically retrieved from multiple electronic databases, including Scopus, Web of Science, PubMed, EBSCOhost, Research4Life, African Journals Online (AJOL), and Google ScholarAdditional sources were identified through citation tracking of selected publications.

The searches were conducted directly within the databases or through the Publish or Perish software (https://harzing.com/resources/publish-or-perish), which was used to query the same databases. The search process took place between 3 July and 5 November 2025, with databases being queried iteratively throughout this period to ensure comprehensive coverage of the literature. Although exact search dates for each database were not recorded, the same search strategy and inclusion criteria were applied consistently across all databases. The search strategy incorporated the use of Boolean operators and truncation to maximize sensitivity and retrieve variations of key terms. The following search string was applied: (“Plant extract*” OR “Botanical*” OR “Biopesticide*”) AND (“Culex*” OR “Aedes*” OR “*Anopheles*” OR “Mosquito*” OR “Vector*”) AND “Tanzania”. For Google Scholar, searches were conducted using the same keywords through Publish or Perish, with results limited to 200 items, which were then screened.

Studies were included if they evaluated live plants or plant extracts obtained from Tanzanian flora for their insecticidal and repellency activity against mosquito vectors found in the country. Both field-based and laboratory-based studies were considered. Studies were excluded if they were not in English, lacked peer review, used imported plant materials, or were unavailable in full text (e.g., abstracts only or non-retrievable). Grey literature was not included in this review, as the focus was restricted to peer-reviewed, full-text articles to ensure data quality and reliability. All retrieved records from different databases were imported into a reference management software (Zotero, version 6.0; https://www.zotero.org/).

### 2.2 Data extraction, management and analysis

The extracted data included the year of publication, geographical region of plant collection, plant family and species, plant part used, study type (field or laboratory), extraction method and solvent type, concentration applied, larval LC₅₀ values, mosquito species and life stages targeted, repellency effects, adult mortality rates, and identified phytochemical constituents. All data were compiled into a Microsoft Excel spreadsheet (Microsoft Corporation, 2019), cleaned to correct omissions and recording errors, and subsequently re-checked for accuracy against the original articles.

Larvicidal activity was evaluated using LC₅₀ values, and extracts were classified based on established thresholds: highly effective (LC₅₀ < 50 μg/mL), effective (50–100 μg/mL), moderate (100–200 μg/mL), weak (200–750 μg/mL), and inactive (>750 μg/mL), following published criteria (Pavela, 2015; Komalamisra et al., 2005; Bücker et al., 2013; Samwel et al., 2022). LC₅₀ was used as the primary metric due to its established cut-off classifications and widespread reporting in larvicidal bioassays. LC₉₀ values were not considered, as no standardized interpretive cut-off classifications exist in the literature. All included studies used standardized larvicidal bioassays; however, variations in exposure time points were observed and are described in the results.

Descriptive statistical analyses were conducted to summarize the data using frequencies and percentages, employing both Microsoft Excel and IBM SPSS® Statistics version 22 (IBM Corp., Armonk, NY, USA). The results were presented using tables, bar charts, and pie charts to facilitate clear interpretation and comparison.

## 3 Results

A systematic literature search following PRISMA guidelines (Fig. 1) identified a total of 163 records, 159 from databases, while 4 additional records were obtained from other sources, including the reference lists of selected articles. After removing 94 duplicates and excluding 15 studies not related to mosquito control, 54 full-text articles were screened. Of these, 13 were further excluded because they used plant extracts sourced from outside Tanzania, despite being tested within the country or involving Tanzanian researchers (see Supplementary File 1). Consequently, 41 studies were retained for inclusion in the final review.

A total of 74 plant species (22 families) were collected from 13 Tanzanian regions, with 46 evaluated for insecticidal activity (see Supplementary File 2). The review captured details such as plant parts used, mainly leaves, roots, and bark, the experimental settings (laboratory or field), extraction methods, and efficacy results, including larvicidal activity indicated by LC₅₀ values. Additionally, repellency rates, adult mosquito mortality, and the presence of bioactive phytochemicals were reported. Full details of this information are presented in the following sections.

### 3.1 Geographic distribution, botanical diversity, plant parts tested, and extraction techniques in the reviewed studies on plant-based mosquito control in Tanzania

**Fig. 2:**
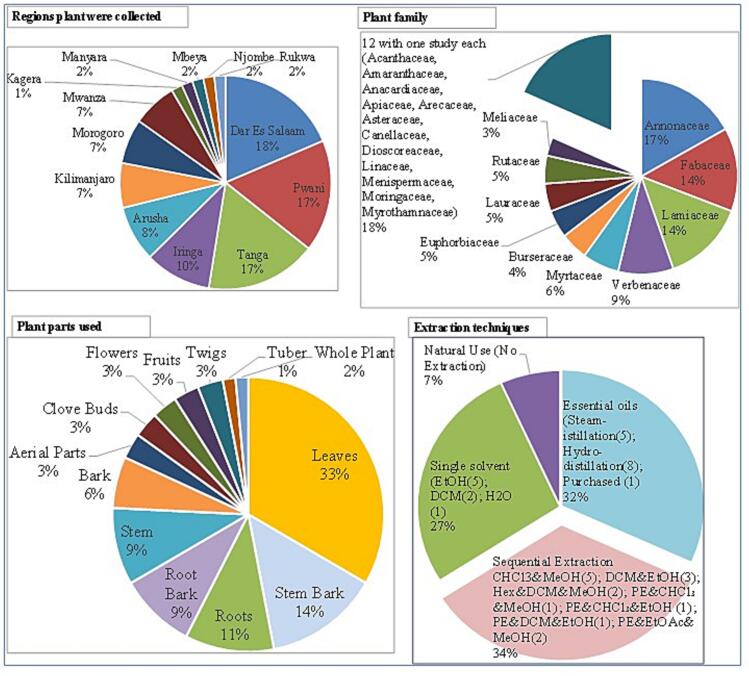
Summary of regions studied, plant families and parts used, and extraction methods applied in the reviewed literature.

The reviewed studies sourced plant materials used in mosquito control trials from 13 regions across Tanzania, with Dar es Salaam (18%), Tanga and Pwani (17% each) being the leading areas of collection. These were followed by Iringa (10%) and Arusha (8%), while Kilimanjaro, Morogoro, and Mwanza contributed moderately (7% each). Regions such as Kagera, Manyara, Mbeya, Njombe, and Rukwa were less frequently involved, each contributing only 1 to 2%. This geographic distribution reflects a concentration of research and plant sourcing in the coastal and northeastern zones of the country, where both biodiversity and access to research institutions are relatively high (Fig. 2).

A total of 22 plant families were represented in the reviewed studies, highlighting the botanical diversity explored for mosquito control. The most frequently cited family was Annonaceae (17%), followed closely by Fabaceae and Lamiaceae (14% each). Other notable families included Verbenaceae (9%), Myrtaceae (6%), Euphorbiaceae, Rutaceae, and Lauraceae (5% each), while 12 families, including Acanthaceae, Apiaceae, and others were represented by a single species each (Fig. 2).

In terms of plant parts utilized, leaves were by far the most common (33%), likely due to their abundance and regenerative properties, supporting sustainable harvesting. Stem bark (14%), roots (11%), root bark and stems (9% each), and bark (6%) were also commonly used. Less frequently used parts included aerial parts, flowers, fruits, twigs, and whole plants, suggesting selectivity based on phytochemical richness and traditional knowledge.

Extraction techniques varied across studies. Sequential solvent extraction using combinations of solvents like petroleum ether, dichloromethane, ethanol, and methanol was the most widely used approach (34%), allowing for fractionation and bioassay-guided isolation. Essential oils, primarily obtained via hydro-distillation or steam distillation, accounted for 32% of the methods and were particularly popular in repellency studies. Single-solvent extractions using ethanol, water, or dichloromethane represented 27%, while a smaller proportion (7%) of studies relied on direct use of plant materials such as burning leaves or applying powders without prior extraction (Fig. 2).

### 3.2 Overview of the characteristics of studies included in the review

**Fig. 3:**
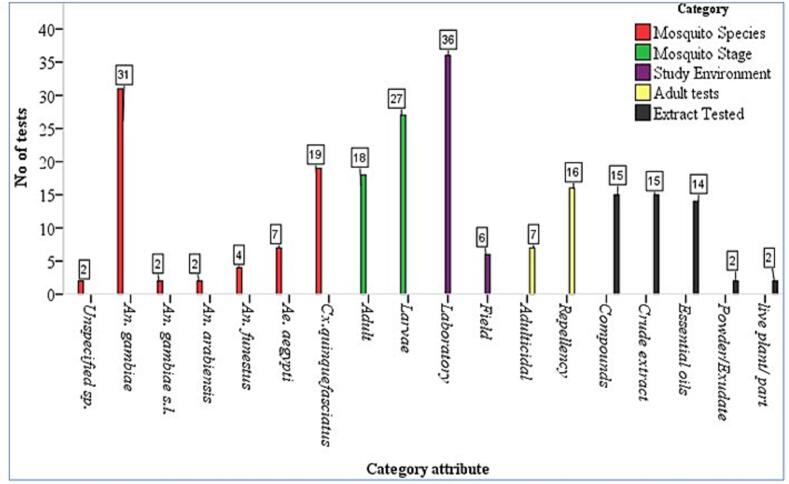
Categorization of key attributes in reviewed studies on plant-based mosquito control.

Mosquito species and stages targeted, experimental settings, control activities evaluated, and types of plant extracts used were assessed across the reviewed literature (Fig. 3). *Anopheles gambiae* was the most frequently targeted species in insecticidal tests (31), followed by *Culex quinquefasciatus* (19), *Aedes aegypti* (7), and other species such as *An. funestus*, *An. arabiensis*, *An. gambiae s.l.*, and unspecified mosquito species, each appearing in 2 to 4 tests. Larvicidal tests were more common (27) than adulticidal and repellent tests (18). Most experiments were conducted under laboratory conditions (36), with relatively few in field settings (6). For adult mosquito control, repellency was evaluated in 16 tests, and adulticidal activity was evaluated in 7 tests. Crude extracts and isolated compounds were the most frequently assessed (15 tests each), followed by essential oils (14 tests). Powder/exudates and live plant/part materials were rarely tested (2 tests each).

### 3.3 Larvicidal activity classification of crude extracts, essential oils, and compounds in the reviewed studies

A total of 55 plant-derived crude extracts, essential oils, and isolated compounds from 32 plant species were evaluated for larvicidal activity against *Anopheles*, *Aedes*, and *Culex* mosquito species (Table 1). Of these, 41.8% (*n* = 23) were classified as *highly effective* (LC₅₀ < 50 μg/mL), 16.4% (*n* = 9) as *effective*, 16.4% (n = 9) as *moderate*, and 21.8% (*n* = 12) as *weak*, with only 3.6% (n = 2) considered *ineffective*, based on classification criteria by Komalamisra et al., (2005), Bücker et al., (2013), Samwel et al., (2022) and Pavela, (2015). Compounds exhibited the highest potency, with 84.6% (11/13) classified as either effective or highly effective, followed by crude extracts at 57.6% (19/33), and essential oils at 22.2% (2/9). Solvent type also influenced activity: ethanol (EtOH) extracts showed the greatest proportion of effective outcomes (72.7%, 8/11), followed by petroleum ether (PE) at 60.0% (3/5), dichloromethane (DCM) at 55.6% (5/9), and methanol (MeOH) with the lowest at 20.0% (1/5).

**Table 1:** Larvicidal activity of plant extracts against mosquito species: LC₅₀ values and bioactivity classifications.

Plant species	Type of extract	Mosquito species	LC_50_ in μg/mL	Classification
(Samwel et al., 2022) *Commiphora merkeri* Engl.	DCM- Arabinofuranosidetridecanols- Compounds	*Ae. aegypti, An. gambiae, Cx. quinquefasciatus*	15.88–40.66*	Highly Effective
(Philbert, 2021) *Dioscorea sansibarensis* Pax	EtOH extract-Crude	*Cx. quinquefasciatus, An. gambiae*	60.9 to 80.7*	Effective
(Daniel et al., 2020) *Kotschya thymodora* (Baker) Dewit & P.A.Duvign.	Vanillic acid: EtOH-(compound)	*Ae. aegypti, Cx. quinquefasciatus, An. gambiae*	62.4	Effective
	Protocatechuic acid: EtOH- Compounds		77.4	Effective
(Samwel et al., 2019) *Commiphora merkeri Engl.*	Natural exudate	*An. gambiae, Ae. aegypti* *Cx. quinquefasciatus*	34.6–41.0*	Highly Effective
	PE-Crude		31.0–38.4*	Highly Effective
	DCM-Crude		10.4–48.0*	Highly Effective
(Sillo et al., 2019) *Hypoestes forskaolii* (Vahl) R.Br.	CHCl₃-Crude	*An. gambiae s.s., Ae. aegypti* *Cx. quinquefasciatus*	2.0–6.0**	Highly Effective
	MeOH-Crude		6.4–11.5**	Highly Effective
	Essential oil: hydro-distillation	*An. arabiensis* (wild)	230	Weak
		*An. gambiae* (lab)	10.5	Highly Effective
*Cinnamomum verum J.Presl*	Essential oil: hydro-distillation	*An. arabiensis* (wild)	130	Moderate
		*An. gambiae* (lab)	20	Highly Effective
(Mkangara et al., 2015) *Commiphora swynnertonii* (Burtt) Engl.	EtOAc-Crude	*An. gambiae* *Cx. quinquefasciatus, Ae. aegypti*	5.8–11.8	Highly Effective
	PE-Crude		3.5–28.9	Highly Effective
	MeOH-Crude		27–228**	Weak
(Kidukuli et al., 2015) *Tephrosia vogelii* Hook.f.	EtOH-Crude	*Anopheles gambiae, Cx. quinquefasciatus.*	20–38*	Highly Effective
(Nkya et al., 2014) *Moringa oleifera* Lam.	EtOAc-Crude	*An. gambiae, Ae. aegypti* *Cx. quinquefasciatus*	87–219**	Weak
	PE-Crude		9.2–513**	Weak
	MeOH-Crude		15.2–366**	Weak
(Nondo et al., 2011) *Cissampelos mucronata A.Rich.*	DCM-Crude	*Cx. quinquefasciatus*	123.0	Moderate
	EtOH-Crude		217.0	Weak
*Tephrosia villosa* Pers.	EtOH-Crude		54.5–169.8	Moderate
(Daniel et al., 2011) *Annona squamosa* L.	EtOH- Crude	*Cx. quinquefasciatus*	9.23–53.25**	Moderate
	Ground P		6.3–37.6**	Highly Effective
(Runyoro et al., 2010) *Ocimum kilimandscharicum* Guerke	Essential oil; hydro-distillation	*Cx. quinquefasciatus*	323.0*	Moderate
*Ocimum basilicum* L.			269.2*	Moderate
*Ocimum suave* Willd.			151.0–170*	Moderate
*Ocimum lamiifolium* Hochst. ex Benth.			229.1*	Moderate
*Artemisia afra* Jacq. ex Willd.			457.1	Weak
(Kihampa et al., 2010a) *Uvariodendron pycnophyllum* (Diels) R.E.Fr	SEQ with CHCl3 and MeOH- Compounds	*An. gambiae*	43–59.0*	Highly Effective
(Kihampa et al., 2010b) *Tessmannia martiniana* Harms	SEQ with CHCl₃ & MeOH- Compounds	*An. gambiae*	15–19	Highly Effective
(Innocent and Magadula, 2010) *Harrisonia abyssinica* Oliv.	DCM-Crude	*Cx. quinquefasciatus*	60.6–150*	Effective
	EtOH-Crude		46.8–74.5*	Effective
(Magadula, 2009) *Annona squamosa* L.	EtOH-Crude	*Cx. quinquefasciatus*	11.0*	Highly Effective
*Annona senegalensis* Pers.			23.4*	Highly Effective
*Annona muricata* L.			56.5*	Highly Effective
(Kihampa et al., 2009a) *Tessmannia densiflora* Harms	SEQ with CHCl₃ & MeOH-Compounds	*An. gambiae*	34–92	Highly Effective
(Kihampa et al., 2009b) *Annona squamosa* L.	SEQ with CHCl₃ & MeOH-Compounds	*An. gambiae s.s.*	20–173	Effective
*Uvaria lungonyana* Verdc.			21–150	Moderate
*Asteranthe lutea* Vollesen			0.5–33.8	Highly Effective
(Innocent et al., 2009) *Synadenium glaucenscen* Pax.	DCM-Crude	*An gambiae*	292.35	Weak
	Hex-Crude		90–109.9	Effective
	MeOH-Crude		538.74	Weak
*Steganotaenia araliacea* Hochst.	DCM-Crude		18.08	Highly Effective
	MeOH- Crude		244.5	Weak
	DCM + VLC: Componds		5.4–10.0	Highly Effective
*Kotschya uguenensis* Verdc.	DCM-Crude		807.54	Infective
*Lantana viburnoides* var. kisi (A.Rich.) Verdc	Hex-Crude		81.84	Effective
(Innocent et al., 2008) *Lantana viburnoides var kisi* (A.Rich.) Verdc	SEQ (Hex, DCM, MeOH); VLC fractions- Crude	*An. gambiae*	7.7–73**	Effective
	SEQ (Hex, DCM, MeOH); VLC fractions-Compounds		5.5–10.4**	Highly Effective
(Nkunya et al., 2004) *Uvaria scheffleri* Diels	PE extract (compounds)	*An. gambiae*	930.5	Ineffective
(Joseph et al., 2004) *Neorautanenia mitis* (A. Rich.) Verdc.	PE extract-Crude	*An. gambiae, Cx. quinquefasciatus*	68*	Highly Effective
	DCM –Crude	*An. gambiae*	110*	Moderate


Activity classification summary		
Activity classification	No. extracts/crude/oils/compounds (*N* = 55)	Percentage
Highly Effective	23	41.8%
Effective	9	16.4%
Moderate	9	16.4%
Weak	12	21.8%
Ineffective	2	3.6%

Proportion of LC₅₀ values classified as highly effective and effective based on the tested extract		
Type Extract	n/N	Percentage
Crude extracts	19/33	57.6%
Essential oils	2/9	22.2%
Compounds	11/13	84.6%

Proportion of LC₅₀ values classified as highly effective and effective based on major extraction solvents		
DCM	5/9	55.6%
PE	3/5	60.0%
EtOH	8/11	72.7%
MeOH	1/5	20.0%

Proportion of LC₅₀ values classified as highly effective and effective based on exposure duration		
24 h	13/18	72.2%
48 h	15/27	55.6%
72 h	4/10	40.0%

Key: Highly Effective = LC_50_ < 50, Effective = 50 < LC_50_ > 100, Moderate =100 > LC_50_ < 200, Weak = 200 > LC_50_ < 750, Ineffective = LC_50_ > 750 (Komalamisra et al., 2005; Bücker et al., 2013; Samwel et al., 2022). Extraction solvents/method key: DCM = Dichloromethane, EtOH = Ethanol, MeOH = Methanol, PE = Petroleum Ether, CHCl₃ = Chloroform, EtOAc = Ethyl Acetate, Hex = Hexane, P = Powder, SEQ = Sequential Extraction, VLC = Vacuum Liquid Chromatography. * = LC_50_ after 24 h, Plain number = after 48 h and ** = after 72 h.

The larvicidal effectiveness of plant extracts varied with exposure time, as different extracts reached effective activity at different intervals. At 24 h, 13 extracts from 10 plant species demonstrated effectiveness, while at 48 h, this increased to 15 extracts from 11 species, with some new species becoming effective. By 72 h, 4 extracts from 2 additional species exhibited larvicidal activity. The effectiveness was influenced by both the plant species and the type of solvent used for extraction, with certain plants showing activity across multiple solvents and time points, while others required longer exposure to become effective (Table 1).

### 3.4 Repellency and adulticidal effects of plant-based products in the reviewed studies

Out of the 41 studies reviewed, 18 (44%) directly assessed repellency and/or mortality effects on adult mosquitoes, yet these provided compelling evidence for the efficacy of plant-based interventions (Table 2). Essential oils were the predominant extract type used in these tests, followed by crude preparations and isolated compounds. *Ocimum* species, particularly *O. suave*, *O. kilimandscharicum*, and *O. gratissimum*, were the most frequently evaluated and demonstrated consistent high repellency (70 to 100%) and moderate adult mortality (47 to 67%) across both laboratory and field assays, including hand-in-cage, tunnel, cone, and human landing tests. Essential oils from *Syzygium aromaticum* (clove) and *Cinnamomum verum* (cinnamon) also showed strong repellency (approximately 88 to 92.7%) and mortality up to 89%, particularly in blends*.* The most potent adulticidal effects, with mortality rates of (95 to 100%), were recorded for plant compounds or extracts from *Warburgia ugandensis*, *Neorautanenia mitis*, *Tessmannia densiflora*, and *Chenopodium ambrosioides*, often applied via treated surfaces or rarely hemolymph injection. Blended essential oils frequently enhanced both repellent and toxic effects. Despite the relatively limited number of studies focusing on adults, these findings highlight the significant role of essential oil extracts, particularly those from *Ocimum* species, in providing promising plant-derived solutions for mosquito vector control.

**Table 2:** Tested plant-derived substances for adult mosquito repellency and mortality.

Study	Plant species	Study type	Extract and test	Species	Adult repellency	Adult mortality
(Emidi, 2025)	*Chenopodium ambrosioides* L.	Laboratory	Essential oil -filter papers	*An. gambiae s.l*., *An. funestus*, *Ae. aegypti*	_	>95%
(Sanga et al., 2023)	*Syzygium aromaticum* (L.) Merr. & L.M.Perry	Laboratory	Essential oils- Tunnel test	*An. gambiae*	∼92.7%	∼77%; ∼86%(blend)
	*Cinnamomum verum* J. Presl			*An. gambiae*	∼88%	89%; 86%(blend)
(Inocente et al., 2019)	*Warburgia ugandensis* Sprague	Laboratory	Compounds-Hemolymph injection	*Ae. aegypti*	_	100%
(Innocent and Hassanali, 2015)	*Lantana viburnoides* (Forssk.) Vahl*, Clausena anisata,*(Willd.) Hook.f. ex Benth. *Uvariodendron gorgonis* (Diels) R.E.Fr	Laboratory	Essential oils-Human landing	*An. gambiae*	High repellence	_
(Leonidas et al., 2015)	*Ocimum suave* Willd.	Laboratory	Essential oils-Hand in cage	*An. gambiae*	High repellency	_
(Innocent et al., 2014)	*Azadirachta indica* A.Juss.*, Annona* spp.*, Ocimum* spp.*, Citrus* spp.*,* and four others	Field-Ethnobotanical survey & participatory approach	Plants (wild, farms, gardens) by burning, soaking, or spraying	Unspecified sp.	High perceived repellence	_
(Suleiman et al., 2014)	*Artabotrys hexapetalus* Hook.f. & Thomson*, Artabotrys rupestris* Engl.	Laboratory	Essential oils- Hand in cage	*An. gambiae*	High repellency	_
(Malebo et al., 2013)	*Ocimum gratissimum L, Ocimum tenuiflorum* L.*, Hyptis suaveolens* (L.) Poit.	Laboratory & Field	Essential oils-Hand in cage, Field human landing	*An. gambia*, *An. funestus, Cx. quinquefasciatus*	70–100% for 5 h	_
(Kweka et al., 2012)	*Ocimum suave* Willd.	Laboratory	Essential oils (blends prepared)-Cage tests & field human landing	*An. gambiae s.l., Cx. quinquefasciatus*	77.6–100% and 58.8–98.8% (blends) for 7 h	_
		Field			82–85% (blend) for 7 h	_
(Mng'ong'o et al., 2011)	*Lantana camara* L.	Field-Ethnobotany	Live plants grown around houses for natural screening.	*An. gambia*	56% for 12 h	_
				*An. funestus*	83% for 12 h	
(Kihampa et al., 2010a)	*Uvariodendron pycnophyllum* (Diels) R.E.Fr	Laboratory	Compounds- Treated bed-net on cage and Hand in cage	*An. gambiae*	∼100%	~ 50%
(Kweka et al., 2010)	*Ocimum kilimandscharicum* Gürke*, Ocimum suave* Willd.	Laboratory	Essential oils-Oviposition deterrence tets	*An. gambiae*	Strong deterrence	_
(Innocent et al., 2010),	*Suregada zanzibariensis* Baill	Laboratory	Essential oils-Hand in cage	*An. gambiae*	∼90%	_
(Kihampa et al., 2009a)	*Tessmannia densiflora* Harms	Laboratory	Compounds (Tessmanic acid)-filter papers	*Anopheles gambiae*	High repellence	100%
(Kweka et al., 2008a)	*Ocimum suave* Willd.	Laboratory and Field	Essential oils-Cone, tunnel test, Hand in cage, Filter paper	*An. gambiae, An. arabiensis, Cx. quinquefasciatus*	83–91%	50–67%
	*Ocimum kilimandscharicum* Gürke				71–92%	47.3–65%
(Kweka et al., 2008b)	*Ocimum suave* Willd.	Laboratory & Field, experimental huts	Essential oils- Human landing and smoking	*An. arabiensis*	91.98% for 4 h	_
	*Ocimum suave* Willd.			*Cx. quinquefasciatus*	88.65% for 4 h	_
	*Ocimum kilimandscharicum* Gürke			*An. arabiensis*	89.75% for 4 h	_
	*Ocimum kilimandscharicum* Gürke			*Cx. quinquefasciatus*	90.50% for 4 h	_
(Malebo et al., 2005)	*Ocimum suave* Willd.	Field & community trials	Essential oils- Hand in a cage, Field human landing	*Anopheles gambiae*	83% for 3 h	_
				*An. funestus*	100% for 3 h	_
				*Cx. quinquefasciatus*	75% for 3 h	_
(Joseph et al., 2004)	*Neorautanenia mitis* (A. Rich.) Verdc.	Laboratory	Compounds-Treated bed-net on a cage	*An. gambiae*	_	∼100%

## 4 Discussion

This review highlights significant progress in plant-based mosquito control research in Tanzania, with 74 plant species identified and 46 tested for insecticidal activity over the past two decades, findings that align with similar studies conducted in South Africa (Maharaj et al., 2012). This reflects growing national interest in indigenous botanical alternatives. However, most studies were concentrated in just 13 of the 31 regions in the county, particularly Dar es Salaam, Tanga, and Pwani. These areas are near key academic institutions such as the University of Dar es Salaam and Muhimbili University of Health and Allied Sciences, where most of the studies were conducted. While this proximity facilitates research, it limits ecological representation. Expanding research to underrepresented regions is essential for capturing a broader diversity of plant chemotypes and varieties, as the potency of extracts can vary significantly with environmental conditions, even within the same species (Innocent and Magadula, 2010; Daniel et al., 2011; Benomari et al., 2023).

The most frequently reported insecticidal plant families for mosquito bioinsecticide tests in Tanzania, as identified in the reviewed articles, were Annonaceae, followed by Fabaceae and Lamiaceae. This contrasts with global trends, where Lamiaceae or Asteraceae dominate, followed by Fabaceae and Rutaceae (Pavela et al., 2019; Pavela, 2015). This highlights the uniqueness of local flora and the importance of using indigenous plant resources to develop context-specific mosquito control tools (Ghosh et al., 2012). Such regional variation supports WHO's call for locally adapted, sustainable vector control strategies (WHO, 2017). Leaves were the most commonly used plant parts in this review, likely due to their abundance, ease of harvesting, and ability to regenerate sustainably. This aligns with previous studies, which cite their high secondary metabolite content and lower ecological impact (Maharaj et al., 2012; Teshome et al., 2025). Although roots and stem bark are often rich in phytochemicals, their use raises sustainability concerns, as they are non-renewable and may lead to overharvesting or plant mortality if not managed appropriately. Moreover, other parts such as flowers and seeds should be considered since their ecological impact of using them is minimal.

Sequential solvent extraction emerged as the most commonly employed method in the reviewed studies, followed by steam or hydro-distillation for essential oils; single-solvent extraction was less frequently used, and the use of fresh plant material was rare. Sequential extraction is considered more efficient, yielding broader and more bioactive profiles than single-solvent techniques (Maria John et al., 2018; Palaiogiannis et al., 2023). While single-solvent extraction may miss some compounds, water and ethanol can still extract key bioactive extracts (Palaiogiannis et al., 2023; Moomin et al., 2023). The predominant use of steam and hydro-distillation for essential oils likely reflects their lower operational costs, despite the higher yields offered by more advanced techniques like supercritical fluid extraction, microwave-assisted extraction, or ultrasound-assisted extraction, which require greater investment (Zhou et al., 2023). Emphasis should be placed on efficient, scalable techniques for plant-based insecticidal development.

Some common biases identified in the designs of the reviewed studies include mosquito species targeted, developmental stages tested, experimental settings, and types of extracts evaluated. Most studies focused on *Anopheles* mosquitoes due to their major role in malaria transmission in sub-Saharan Africa (WHO, 2024a), while *Aedes* and *Culex* species received comparatively less attention, despite their increasing involvement in arboviral outbreaks, potentially limiting preparedness for emerging vector-borne threats. Insecticidal testing was largely conducted on larvae, as larval control is considered more proactive, target-specific, environmentally friendly, and cost-effective for preliminary screening (Ghosh et al., 2012; Piplani et al., 2019). This methodological variability limits the direct comparability of findings across studies. Nevertheless, this variability does not detract from the purpose of the review, which was to synthesize and map the existing evidence and provide a descriptive overview, rather than to undertake a quantitative quality assessment or statistical analysis as is typically done in meta-analyses.

A key limitation identified in this review is the dominance of laboratory-based studies, with more than 90% conducted under controlled conditions. Although laboratory experiments are crucial for preliminary screening, they often fail to capture the complexity of real-world environments. The limited translation of laboratory findings into field applications is largely attributed to the inherent instability of phytochemicals (Lahlali et al., 2022). Plant-derived compounds, including crude extracts, essential oils, and isolated constituents are highly susceptible to degradation by environmental factors such as light, temperature, oxygen, and pH (Lahlali et al., 2022). Essential oils are particularly vulnerable due to their volatility and tendency to oxidize, which significantly reduces their persistence and effectiveness in open environments (Ayllón-Gutiérrez et al., 2024; Gupta et al., 2023). While biodegradability is an environmental advantage, it results in short residual activity and necessitates frequent reapplication, which may limit operational feasibility (Şengül Demirak and Canpolat, 2022). These challenges are not unique to Tanzania; global studies have similarly reported that only a small proportion of plant-based insecticides progress beyond laboratory evaluation due to issues related to stability, formulation, and field validation (Pavela et al., 2019; Isman, 2020). Evidence from other African contexts, including South Africa, further demonstrates that crude plant extracts often exhibit reduced and inconsistent efficacy under field conditions (Maharaj et al., 2012; Maharaj et al., 2011), reinforcing the need for improved formulation strategies (Maia and Moore, 2011).

To overcome these limitations, recent research has increasingly focused on innovative formulation approaches designed to enhance the stability and efficacy of plant-based insecticides. Techniques such as nanoemulsions, microencapsulation, and polymer-based delivery systems can protect active compounds from environmental degradation, reduce volatility, and enable controlled release (Pavela et al., 2019; Ayllón-Gutiérrez et al., 2024; Gupta et al., 2023). These advances have shown promising results in semi-field and applied studies, where improved persistence and bioactivity have been observed (Pavela et al., 2019; Maia and Moore, 2011; Lahlali et al., 2022; Şengül Demirak and Canpolat, 2022). However, the application of such technologies remains limited in many African settings due to financial and technical constraints. Bridging this gap will require targeted investment, capacity building, and adaptation of these technologies to local contexts.

In relation to the plant extracts identified in this review, crude extracts, essential oils, and isolated compounds were tested in nearly equal proportions, suggesting noteworthy progress in Tanzania's research toward the evaluation of isolated phytochemical compounds. This pattern aligns with the global trend of increasing emphasis on isolated compounds in plant-based insect control research (Turchen et al., 2020).

This review evaluated the larvicidal potency of 55 plant extracts for 32 species of plants by classifying their efficacy based on established LC₅₀ thresholds, comparing extract types and solvents, highlighting how rapidly effective extracts act, and identifying plant species with potential for the development of plant-based larvicides. The classification followed LC₅₀ cut-off values proposed in the literature, with concentrations below 50 μg/mL and 100 μg/mL considered highly effective and effective, respectively (Pavela, 2015; Komalamisra et al., 2005). More than half of the tested extracts met these efficacy criteria under laboratory conditions, indicating significant potential for further field evaluation. A majority of the active extracts were isolated compounds, whereas crude extracts and essential oils showed comparatively lower potency, which is consistent with previous studies reporting the superior bioactivity of purified phytochemicals (Pavela et al., 2019; Ghosh et al., 2012). Among extraction solvents, EtOH extracts exhibited the highest larvicidal efficacy compared to PE, DCM, and MeOH, a trend attributed to EtOH's ability to extract a broader spectrum of bioactive secondary metabolites such as alkaloids, flavonoids, and terpenoids (Palaiogiannis et al., 2023; Baz et al., 2024). Additionally, EtOH is favored for its safety, ease of handling, and the absence of surfactant requirements during bioassay preparations (Pavela et al., 2019; Patel et al., 2021).

Out of 32 plant species tested for larvicidal activity, 17 demonstrated rapid and highly effective activity within 24 to 48 h, including *Commiphora merkeri*, *Dioscorea sansibarensis*, *Tephrosia vogelii*, *Uvariodendron pycnophyllum*, *Harrisonia abyssinica*, *Annona squamosa*, *Annona senegalensis*, *Annona muricata*, *Neorautanenia mitis*, *Kotschya thymodora*, *Syzygium aromaticum*, *Cinnamomum verum*, *Commiphora swynnertonii*, *Tessmannia martiniana*, *Tessmannia densiflora*, *Asteranthe lutea*, and *Steganotaenia araliacea*. Their fast-acting larvicidal effects are due to the presence of potent phytochemicals such as rotenone, terpenoids, acetogenins, and phenolics, which disrupt mosquito larvae through neurotoxic and respiratory pathways (Chaudhary et al., 2024). Given their high efficacy, these species are strong candidates for field trials to assess scalability, environmental persistence, formulation stability, and ecological safety.

Despite limited research on adult mosquitoes, plant-based interventions, especially essential oils, show strong repellency and adulticidal potential. *Ocimum* species (*O. suave*, *O. kilimandscharicum*, *O. gratissimum*) consistently exhibit high repellency and moderate mortality. Similarly, essential oils from *Syzygium aromaticum* and *Cinnamomum verum* demonstrate strong repellency and enhanced mortality, particularly when used in blends, suggesting synergistic effects. Extracts from *Warburgia ugandensis*, *Neorautanenia mitis*, *Tessmannia densiflora*, and *Chenopodium ambrosioides* achieve the highest adulticidal activity (95–100% mortality). Although biopesticide products derived from *Ocimum* species, *Syzygium aromaticum*, *Cinnamomum verum*, and *Chenopodium ambrosioides* have been developed and commercialized for insect control (Khursheed et al., 2022; Pavela, 2016), developing products from locally available flora remains crucial to enhance accessibility, reduce costs, and ensure their field efficacy. To fully harness this potential, future work should focus on improving formulation stability and validating effectiveness through comprehensive field trials.

While advanced technologies, including formulation approaches such as nanoformulations and microencapsulation, can enhance the stability and efficacy of plant-based insecticides (Pavela et al., 2019; Ayllón-Gutiérrez et al., 2024; Gupta et al., 2023), and modern extraction methods such as supercritical fluid, microwave-assisted, and ultrasound-assisted techniques can increase yields and broaden the diversity of bioactive compounds (Zhou et al., 2023; Zhang et al., 2018) their application in Tanzania and sub-Saharan Africa remains constrained by cost, technical complexity, and limited accessibility. As a result, the use of these approaches in low-resource settings is often not feasible. Consequently, vector control strategies should prioritise cost-effective, scalable, and locally adaptable approaches based on readily available pesticidal plants.

As highlighted by Isman (2017), the effective application of botanical insecticides in African settings depends on simple and practical methods. At the household level, aqueous plant extracts, often enhanced with basic adjuvants such as soap, can be prepared and applied with minimal resources. At the community level, cooperative approaches can improve scalability, consistency, and knowledge sharing. Therefore, optimising the use of known bioactive plant species through locally feasible methods may be more impactful than focusing solely on the discovery of new species. This aligns with the findings of the present review, which highlight the availability of effective indigenous plants and the importance of context-specific, sustainable mosquito control strategies (Pavela et al., 2019; Şengül Demirak and Canpolat, 2022). Thus, although advanced technologies remain important for future development, priority should be given to cost-effective and locally adaptable solutions for sub-Saharan Africa.

## 5 Future directions

To advance the development of plant-based bio-insecticides for mosquito control in Tanzania and in bordering countries in east Africa, future research should focus on several key priorities. Plant species such as *Commiphora merkeri*, *Dioscorea sansibarensis*, *Tephrosia vogelii*, *Neorautanenia mitis*, *Warburgia ugandensis*, *Syzygium aromaticum*, and *Cinnamomum verum* demonstrate strong bioactivity and should be prioritised for further development. Expanding studies into underexplored regions is essential to capture broader plant diversity and environmental influences on bioactivity. Given the predominance of laboratory-based research, well-designed field trials are needed to assess efficacy, persistence, and environmental safety under operational conditions. In addition, improving formulation strategies, including nanoemulsions and microencapsulation, and exploring synergistic combinations of plant extracts or with low-dose synthetic insecticides may enhance efficacy while reducing chemical use (Pavela et al., 2019; Ahmed et al., 2022). Practical implementation should emphasise low-cost, locally adaptable approaches, such as simple aqueous extracts for household use and community-based strategies to improve scalability and knowledge sharing. Finally, standardisation of extraction methods, bioassay protocols, and reporting metrics is essential to improve comparability and support translation into practical applications.

## 6 Conclusion

This review highlights significant progress in plant-based mosquito control research in Tanzania, identifying numerous indigenous plant species with potent larvicidal and adulticidal properties. However, most studies are concentrated near academic institutions, limiting ecological diversity and overlooking unique plant chemotypes from underexplored regions. Additionally, a strong reliance on laboratory-based bioassays and conventional extraction methods hinders the translation of promising results into effective field applications. To maximize the potential of Tanzania's rich botanical resources, future research should expand geographically, utilize advanced extraction and formulation technologies, and focus on comprehensive field trials to ensure real-world efficacy and scalability. Developing eco-friendly, locally sourced mosquito control products will improve accessibility, reduce costs, and support global efforts for sustainable and context-specific vector management, which is critical in addressing malaria and the rising threat of mosquito-borne diseases in Tanzania and sub-Saharan Africa.

## CRediT authorship contribution statement

**Deokary Joseph Matiya:** Writing – review & editing, Writing – original draft, Visualization, Validation, Supervision, Software, Resources, Project administration, Methodology, Investigation, Funding acquisition, Formal analysis, Data curation, Conceptualization.

## Funding

This work was supported by the Dar es Salaam University College of Education.

## Declaration of competing interest

The author declare that they don't have any conflict of interest.

## Appendix A Supplementary data

Supplementary File 1 Image 1

Supplementary File 2 Image 2
