# Supplementary material for: Prospection of plant-based bio-insecticides for mosquito vector control in Tanzania: A comprehensive review
Source: Parasite Epidemiol Control. 2026 Apr 17;33:e00505. doi: 10.1016/j.parepi.2026.e00505 (PMC13101672; doi:10.1016/j.parepi.2026.e00505)
Supplement: Supplementary file 1 [file mmc1.docx]

Table S1. Excluded full-text articles that tested the plant extract against mosquitoes, and the reasons for exclusion.

| **Citation** | **Reason for exclusion** |
| --- | --- |
| [1] | Plant material (*Aframomum angustifolium* and *Tagetes patula*) was evaluated in Tanzania, although it was originally sourced from Mbarara, Uganda. |
| [2] | The plant species (*Ocimum canum*) is not native to Tanzania; however, three of the researchers are from Tanzania. |
| [3] | Plant material (*Feronia limonia*) was evaluated in Tanzania, although it was originally sourced from Tamil Nadu, India. |
| [4] | Plant material (*Pelargonium roseum* and*Juniperus virginiana*) was evaluated in Tanzania, although it was originally sourced from Tehran, Iran. |
| [5]) | The plant species (*Glycosmis pentaphylla*) is not native to Tanzania; however, one of the researchers is from Tanzania. |
| [6] | Plant material (*Cryptomeria japonica*) was evaluated in Tanzania, although it was originally sourced from Antou, Taiwan. |
| [7] | Plant material (*Cinnamomum osmophloeum*) was evaluated in Tanzania, sourced from Antou, Taiwan. |
| [8] | Plant material (*Vitex trifolia* leaves, *Vitex schiliebenii*, and *Vitex payos*) was sourced from the Kenyan coastal region, while the mosquitoes tested were from Tanzania. |
| [9] | Plant material (*Plectranthus amboinicus*) was evaluated in Tanzania; the material was sourced from Tamil Nadu, India. |
| [10] | lant material (*Schinus terebinthifolia*) was evaluated in Tanzania; the material was sourced from Vitória, Espírito Santo, Brazil. |
| [11] | Plant material (*Ocimum kilimandscharicum*) was evaluated in Tanzania; the material was sourced from Naivasha, Kenya. |
| [12] | Plant-derived chemicals sourced from various manufacturers in the USA, UK, and Europe were evaluated on mosquitoes collected in Tanzania. |
| [13] | Plant-derived chemicals extracted from *Eucalyptus maculata citriodora*, sourced from manufacturers in the UK, were evaluated in Tanzania. |

**References**

[1] A. Thomas, E.J. Kweka, P.E. Ogwang, Laboratory and simulated semi-field larvicidal efficacy of *Aframomum angustifolium* (Sonn.) K. Schum and *Tagetes patula* essential oils against *Anopheles gambiae*, Journal of Natural Pesticide Research 7 (2024) 100067. https://doi.org/10.1016/j.napere.2024.100067.

[2] J.M. Murugan, G. Ramkumar, B. Mwang’onde, A. Thomas, M.J. Mihale, R. Muthusamy, M.S. Shivakumar, E.J. Kweka, Isolation, characterization and docking analysis of insecticidal compound from *Ocimum canum* methanolic leaf extracts and its potential against three mosquito vectors, Journal of Natural Pesticide Research 7 (2024) 100062. https://doi.org/10.1016/j.napere.2023.100062.

[3] E.J. Kweka, F.P. Mdoe, N.N. Lowassari, V. Venkatesalu, A. Senthilkumar, The Laboratory and Semi-Field Larvicidal Effects of Essential Oil Extracted from *Feronia limonia* against *Anopheles arabiensis* Patton, J Parasitol Res 2023 (2023) 5907603. https://doi.org/10.1155/2023/5907603.

[4] R. Yohana, P.S. Chisulumi, W. Kidima, A. Tahghighi, N. Maleki-Ravasan, E.J. Kweka, Anti-mosquito properties of *Pelargonium roseum* (Geraniaceae) and *Juniperus virginiana* (Cupressaceae) essential oils against dominant malaria vectors in Africa, Malar J 21 (2022) 219. https://doi.org/10.1186/s12936-022-04220-8.

[5] G. Ramkumar, S. Karthi, R. Muthusamy, P. Suganya, D. Natarajan, E.J. Kweka, M.S. Shivakumar, Mosquitocidal Effect of *Glycosmis pentaphylla* Leaf Extracts against Three Mosquito Species (Diptera: Culicidae), PLoS ONE 11 (2016) e0158088. https://doi.org/10.1371/journal.pone.0158088.

[6] F.P. Mdoe, S.-S. Cheng, L. Lyaruu, G. Nkwengulila, S.-T. Chang, E.J. Kweka, Larvicidal efficacy of *Cryptomeria japonica* leaf essential oils against *Anopheles gambiae*, Parasites Vectors 7 (2014) 426. https://doi.org/10.1186/1756-3305-7-426.

[7] F.P. Mdoe, S.-S. Cheng, S. Msangi, G. Nkwengulila, S.-T. Chang, E.J. Kweka, Activity of *Cinnamomum osmophloeum* leaf essential oil against *Anopheles gambiae* s.s, Parasit Vectors 7 (2014) 209. https://doi.org/10.1186/1756-3305-7-209.

[8] M.G. Nyamoita, I. Ester, M.H. Zakaria, L. Wilber, O.J. Bwire, H. Ahmed, Comparison of the effects of extracts from three *Vitex* plant species on *Anopheles gambiae* s.s. (Diptera: Culicidae) larvae, Acta Tropica 127 (2013) 199–203. https://doi.org/10.1016/j.actatropica.2013.05.003.

[9] E.J. Kweka, A. Senthilkumar, V. Venkatesalu, Toxicity of essential oil from Indian borage on the larvae of the African malaria vector mosquito, *Anopheles gambiae*, Parasit Vectors 5 (2012) 277. https://doi.org/10.1186/1756-3305-5-277.

[10] E.J. Kweka, M. Nyindo, F. Mosha, A.G. Silva, Insecticidal activity of the essential oil from fruits and seeds of *Schinus terebinthifolia* Raddi against African malaria vectors, Parasit Vectors 4 (2011) 129. https://doi.org/10.1186/1756-3305-4-129.

[11] E.J. Kweka, H.M. Nkya, L. Lyaruu, E.E. Kimaro, A.M. Mahande, Efficacy of *Ocimum kilimandscharicum* plant extracts after four years of storage against *Anopheles gambiae* ss, Journal of Cell and Animal Biology 3 (2009) 171–174. http://www.academicjournals.org/JCAB.

[12] E. Innocent, N.K. Gikonyo, M.H. Nkunya, Repellency property of long chain aliphatic methyl ketones against *Anopheles gambiae* s.s, Tanzania Journal of Health Research 10 (2008) 50–54. https://doi.org/10.4314/thrb.v10i1.14342.

[13] J.K. Trigg, Evaluation of a eucalyptus-based repellent against *Anopheles* spp. in Tanzania, J Am Mosq Control Assoc 12 (1996) 243–246.
