# Supplementary material for: Prospection of plant-based bio-insecticides for mosquito vector control in Tanzania: A comprehensive review
Source: Parasite Epidemiol Control. 2026 Apr 17;33:e00505. doi: 10.1016/j.parepi.2026.e00505 (PMC13101672; doi:10.1016/j.parepi.2026.e00505)
Supplement: Supplementary file 2 [file mmc2.docx]

Table S2. Summary of information extracted from the reviewed articles that met the selection criteria.

| **Authors** | **Year** | **Region collected** | **Plant species** | **Plant part used** | **Study type** | **Extraction method & type** | **Concentration dose & Larval LC50** | **Mosquito species & stages** | **Repellency %** | **Adult Mortality rate** | **Phytochemicals identified** |
| --- | --- | --- | --- | --- | --- | --- | --- | --- | --- | --- | --- |
| [1] | 2025 | Tanga | *Chenopodium ambrosioides* L. | Leaves | Laboratory (WHO bioassays) | Essential oil by hydro-distillation; applied on WHO filter papers | 10% solution; LC50 not reported | Adult female *An. gambiae* s.l., *An. funestus*, *Ae. aegypti* | _ | > 95% | Not analyzed |
| [2] | 2021 | Mwanza | *Syzygium aromaticum* (L.) Merr. & L.M.Perry | Clove buds, bark | Laboratory (Tunnel test) | Essential oils by hydro-distillation; impregnated on nets | 45, 55, 65 mg/m²; LC50 not reported | Adult female *An*. *gambiae s.s.,* 2–5 days old | ̴ 92.7%; Cinnamon up to 88% | ̴ 89% cinnamon; ̴ 77% clove; blends ̴ 86% | Clove: Eugenol, phenol, caryophyllene; Cinnamon: Eugenol, β-linalool |
|  |  |  | *Cinnamomum verum* J. Presl |  |  |  |  |  |  |  |  |
| [3] | 2022 | Arusha | *Commiphora merkeri* Engl. | Stem bark exudate | Laboratory (WHO larvicidal) | DCM extract; isolation of compound arabinofuranosidetridecanols | (LC50, ug/mL)  Compound 1 15.88 -40.66; Compound 2 17.7 - 33.79 | Larvae of *Ae. aegypti, An. gambiae*, *Cx. quinquefasciatus* | _ | _ | Arabinofuranosidetridecanols (compounds 1 & 2) |
| [4] | 2021 | Dar es Salaam | *Dioscorea sansibarensis* Pax | Leaves | Laboratory (WHO larvicida) | EtOH leaf extract by maceration and evaporation- Crude | 24hrs (LC50, μg/mL ), 80.70 (*An*), 60.92(*Cx*) | All instar larvae of *An. gambiae* s.s., *Cx. quinquefasciatus* | _ | _ | Not analyzed |
| [5] | 2020 | Njombe | *Kotschya thymodora* (Baker) Dewit & P.A. Duvign. | Leaves | Laboratory (WHO larvicida) | 20% aq. EtOH extract; isolation via VLC –Compounds | 48hrs (LC_50_,μg/mL)  Vanillic acid, 62.  Protocatechuic acid, 77.41 | 3rd instar larvae of *An. gambiae* s.s. | _ | _ | Vanillic acid, protocatechuic acid |
| [6] | 2019 | Manyara | *Hypoestes forskaolii* R.Br. | Roots | Laboratory (WHO larvicidal) | CHCl₃ & MeOH extracts by sequential maceration – Crude | 72hrs (LC_50_, μg/mL) CHCl_3_ 2.03–6.00; MeOH 6.43–11.54. | 3rd instar larvae of  *An. gambiae*, *Ae. aegypti*, *Cx. quinquefasciatus* | _ | _ | Not analysed, but it has 102 metabolites incl. caryophyllene, caryophyllene oxide (from literature) |
| [7] | 2019 | Dar es Salaam | *Commiphora merkeri* Engl. | Stem bark exudate | Laboratory (WHO larvicidal) | Crude exudate; PE & DCM fractions - Crude | LC50 24–72hours(LC_50_,μg/mL): Exudate 24–41; PE 14.9–38.4; DCM 8.8–48.0 | Late  3rd instar larvae of *Ae. aegypti, Cx. quinquefasciatus,*  *An. gambiae* | _ | _ | Not analyzed; actives suggested in the DCM fraction |
| **Study** | **Year** | **Region collected** | **Plant species** | **Plant part used** | **Study type** | **Extraction method & type** | **Concentration dose & Larval LC50** | **Mosquito species & stages** | **Repellency %** | **Adult Mortality rate** | **Phytochemicals identified** |
| [8] | 2019 | Arusha | *Warburgia ugandensis* Sprague | Stem bark | Laboratory (larvicidal & adulticidal) | DCM extract; applied to larvae & adults- Compounds | 100 µM | *Ae. aegypti* larvae and adults | _ | ̴ 100% | Warburganal, polygodial (sesquiterpene dialdehydes) |
| [9] | 2017 | Mwanza | *Syzygium aromaticum* (L.) Merr. & L.M.Perry | Clove bud, bark | Laboratory (WHO larvicidal) | Essential oils purchased raw; by GC-MS | Wild (LC_50_,μg/mL): Clove 159–246; Cinnamon 115–131; Lab: Clove 8.6–19.6; Cinnamon 10.5–12.3 µg/mL | 3rd instar larvae of the lab  *An. gambiae* s.s. & wild *An. arabiensis* | _ | _ | Cinnamon: Eugenol (96.5%), β-Linalool; Clove: Eugenol (99.14%) |
|  |  |  | *Cinnamomum verum* J. Presl |  |  |  |  |  |  |  |  |
| [10] | 2015 | Arusha | *Commiphora swynnertonii* Engl. | Stem bark | Laboratory (WHO larvicidal) | Sequential extraction; PE, EtOAc, MeOH- Crude | 24–72hrs (LC_50_ µg/mL) PE  *Anopheles* 10-485, *Culex*. 3-28µg/mL, *Aedes* 2-25,  EtOAc LC50s: *Anopheles* 8-27, *Culex.* 5-25, *Aedes*  4-14; MeOH LC50; *Anopheles* 238-709, *Culex* 87-887, *Aedes* 27-1236 | Late 3rd instar larvae of *An. gambiae* s.s*., Cx. quinquefasciatus, Ae. aegypti* | _ | _ | Not analyzed; attributed to medium-polar metabolites. |
| [11] | 2015 | Dar es Salaam | *Ocimum suave* Willd. | Leaves | Laboratory (human landing assays) | Essential oil by hydro-distillation (0.2% yield) | RC50: 0.116 mg/cm²; RC75: 0.282; RC90: 0.432; RC99: 0.989 | Adult  *An. gambiae females* (5–9 days old) | High repellency | _ | Eugenol, methyl eugenol, linalool, cis-β-ocimene, linalyl acetate (from previous reports) |
| [12] | 2015 | Pwani, Tanga, Rukwa,, Morogoro | *Tephrosia vogelii* Hook.f. | Leaves, stems, fruits, twigs | Laboratory (WHO larvicidal bioassays) | EtOH extracts from dried, powdered plant parts- Crude | 24-72hrs (LC_50_,μg/mL): LC50s for T. vogelii leaves: *(Anopheles)* 94.77, (*Culex*) 19.7, (*Anopheles.*) 235, (*Culex*) 55 | Late 3rd/early 4th instar larvae of *An. gambiae and*  *Cx. quinquefasciatus* | _ | _ | Rotenone suggested as key component (not analyzed) |
|  |  |  | *Tephrosia villosa* Pers. |  |  |  |  |  |  |  |  |
|  |  |  | *Tephrosia aequilata* Baker |  |  |  |  |  |  |  |  |
|  |  |  | *Tephrosia pumila* Baker |  |  |  |  |  |  |  |  |
| **Study** | **Year** | **Region collected** | **Plant species** | **Plant part used** | **Study type** | **Extraction method & type** | **Concentration dose & Larval LC50** | **Mosquito species & stages** | **Repellency %** | **Adult Mortality rate** | **Phytochemicals identified** |
| [13] | 2015 | Iringa, Dar es Salaam | *Lantana viburnoides* (Forssk.) Vahl  *Clausena anisata* (Willd.) Hook.f. ex Benth.  *Uvariodendron gorgonis* (Diels) R.E.Fr | Leaves | Laboratory (human landing assays) | Essential oils by hydro-distillation | RC50s: *U. gorgonis* 0.38×10⁻⁵ g/cm²; *L. viburnoides* 0.56×10⁻⁵; C. anisata 1.1×10⁻⁵ | Adult female *An. gambiae* s.s. | High repellency | _ | *U. gorgonis*: eugenol, limonene; *L. viburnoides*: piperitenone, artemisia ketone; *C. anisata*: estragol |
| [14] | 2014 | Pwani, Iringa | *Artabotrys hexapetalus* Hook.f. & Thomson | Leaves & stem bark | Laboratory (human landing assays) | Essential oils by fractional hydrodistillation | RC_50_s in mg/cm² _;_ *A. hexapetalus* leaves : 1.81–2.74×10⁻⁵ ; *A. hexapetalus* stem 1.89–7.47×10⁻³; *A. rupestris* stem 1.03–4.81×10⁻³ | Adult female *An. gambiae* s.s. | High, moderate repellency | _ | β-caryophyllene oxide, cadinol, cubenol, (-)-spathulenol, 7-methyl-4-methylene-1-(1-methylethyl)-naphthalene |
|  |  |  | *Artabotrys rupestris* Engl. |  |  |  |  |  |  |  |  |
| [15] | 2014 | Tanga | *Moringa oleifera* Lam. | Flowers | Laboratory (WHO larvicidal & brine shrimp assays) | Sequential extraction: PE, EtOAc, MeOH- Crude | 24-72hrs (LC_50_,μg/mL) ranges: Petroleum ether 9.22–859; EtOAc 15.82–500; MeOH 175.47–513.156 (*An. gambiae, Ae. aegypti, Cx. quinquefasciatus*) respectively | Late 3rd instar larvae of *An. gambiae, Ae. aegypti*, *Cx. quinquefasciatus* | _ | _ | β-sitosterol, β-amyrin, 9-octadecenoic acid suggested from prior reports |
| **Study** | **Year** | **Region collected** | **Plant species** | **Plant part used** | **Study type** | **Extraction method & type** | **Concentration dose & Larval LC50** | **Mosquito species & stages** | **Repellency %** | **Adult Mortality rate** | **Phytochemicals identified** |
| [16] | 2014 | Pwani | *Azadirachta indica* A.Juss.  *Annona spp.*  *Ocimum spp.*  *Citrus spp.*  *Anacardium occidentale* L.  *Mangifera indica* L.  *Psidium spp.*  *Cocos nucifera* L. | Mostly leaves & roots (varies by plant) | Ethnobotanical survey & participatory appraisal | Plants collected from wild, farms, home gardens; applied by burning, soaking, or spraying- No-extraction | Not quantified; community practice descriptions only | Adult mosquitoes (general; not species-specific) | High repellency | _ | Literature supports insecticidal activity of azadirachtin (neem), eugenol (*Ocimum*), limonene (Citrus), and others |
| [17] | 2013 | Pwani,  Morogoro | *Artabotrys modestus* L. | Stem & root bark | Laboratory (WHO larvicidal) | Sequential extraction: PE, CH₂Cl₂, EtOH; compounds isolated by chromatography- Copound | (LC_50_, µg/mL): 1–111 (24h), 2.3–27 (48h), <1–21.9 (72h) for active compounds | Late 3rd/early 4th instar *An. gambiae s.s.* | _ | _ | Artamodamide, artamenone, artamonteirine, karatavin, laudanine, catechin, polycarpol, stigmasterol, others |
|  |  |  | *Artabotrys monteiroae* Oliv. |  |  |  |  |  |  |  |  |
| [18] | 2013 | Tanga  Dar es Salaam | *Ocimum gratissimum* L. | Leaves & aerial parts | Laboratory & field repellency assays | Essential oils by hydrodistillation | RC_50_s (×10⁻⁵ mg/cm²): *O. gratissimum* 2.0, *O. tenuiflorum 8*.0*, H. suaveolens* 15.0 | Adult *An. gambiae* s.s., *An. funestus*, *Cx*. *quinquefasciatus* | 10–30% *O. gratissimum* gave 70–100% protection; 30% *O. gratissimum* >15% DEET | _ | Camphor, eugenol, linalool, cineole, caryophyllene, and 20+ others |
|  |  |  | *Ocimum tenuiflorum* L. |  |  |  |  |  |  |  |  |
|  |  |  | *Hyptis suaveolens* (L.) Poit. |  |  |  |  |  |  |  |  |
| [19] | 2012 | Arusha, Kilimanjaro | *Ocimum suave* Willd. | Leaves (O. suave) | Laboratory cage assays & field human landing catches | Essential oil by steam distillation; blends prepared | Single doses: 10, 20, 30% v/v; blends: MR08+DEET, MR08+*O. Suave*, DEET+*O. suave* | Adult *An. gambiae s.l., Cx. quinquefasciatus* | Lab: 77.6–100%(single), 58.8–98.8% (blend); Field: DEET 88.2%, blends 82–85% | _ | Active ingredients of O. suave cited from prior work |
| **Study** | **Year** | **Region collected** | **Plant species** | **Plant part used** | **Study type** | **Extraction method & type** | **Concentration dose & Larval LC50** | **Mosquito species & stages** | **Repellency %** | **Adult Mortality rate** | **Phytochemicals identified** |
| [20] | 2011 | Pwani | *Cissampelos mucronata* A.Rich. | Roots, aerial parts, leaves, twigs, fruits | Laboratory (WHO larvicidal | Sequential extraction (C. mucronata: DCM & EtOH; T. villosa: EtOH)- Crude | 24 -72 hrs (LC50: μg/mL): *C. mucronata* roots LC50: 117 - 219.1  *T. villosa*; fruits 53.3 – 66; leaves 161.6 – 213, Twig 71.2 – 126.2, Roots 71.5 – 218.5. | Late  3rd instar larvae of  *Culex quinquefasciatus* | _ | _ | Not determined; suggested presence of bioactive larvicidal compounds |
|  |  |  | *Tephrosia villosa* Pers. |  |  |  |  |  |  |  |  |
| [21] | 2011 | Kagera | *Lantana camara* L. | Whole plant (grown around houses) | Field pilot study (household-based) | Live plants grown around houses for natural screening- No extraction | Not expressed as LC50; ~25–150 cm plant height around homes | Adult  *An. gambiae s.s.*, *An*. *funestus* s.s., other mosquitoes | *An. gambiae* s.s. reduced by 56%; *An. funestus s.s.* by 83% | _ | Caryophyllene, eucalyptol, α-humulene, germacrene (from *Lantana* spp. on literature) |
| [22] | 2011 | Various regions | *Neorautanenia mitis* (A.Rich.) Verdc. | Leaves, stems, roots, bark (varies by plant) | Laboratory (larvicidal, tarsal contact, repellency bioassays) | Sequential extraction (DCM, EtOH, others); isolation of pure- compounds | 72hrs (LC_50_, μg/ml) ranges: 5–59 for several isolated compounds; crude extracts varied widely  (A review) | Late 3rd/early 4th instar larvae of *An. gambiae* s.s. | _ | _ | Neorautanone, neoduline, 4-methoxyneoduline, 2',3'-epoxyasteranthine, cleistenolide, tessmannic acid, O-methyleugenol, camaric acid, betulinic acid |
|  |  |  | *Asteranthe lutea* Verdc. |  |  |  |  |  |  |  |  |
|  |  |  | *Cleistochlamys kirkii* (Benth.) Oliv. |  |  |  |  |  |  |  |  |
|  |  |  | *Uvaria scheffleri* Diels |  |  |  |  |  |  |  |  |
|  |  |  | *Tessmannia spp.* |  |  |  |  |  |  |  |  |
|  |  |  | *Uvariodendron pycnophyllum* (Diels) R.E.Fr |  |  |  |  |  |  |  |  |
|  |  |  | *Lantana viburnoides* (Forssk.) Vahl |  |  |  |  |  |  |  |  |
|  |  |  | *Kotschya uguenensis* Verdc. |  |  |  |  |  |  |  |  |
|  |  |  | *Neorautanenia mitis* (A.Rich.) Verdc. |  |  |  |  |  |  |  |  |
| **Study** | **Year** | **Region collected** | **Plant species** | **Plant part used** | **Study type** | **Extraction method & type** | **Concentration dose & Larval LC50** | **Mosquito species & stages** | **Repellency %** | **Adult Mortality rate** | **Phytochemicals identified** |
| [23] | 2011 | Mwanza, Dar es salaam, Kilimaanjaro, Morogoro | *Annona squamosa* L. | Leaves (EtOH extracts & powders) | Laboratory (WHO larvicidal bioassays) | EtOH- crude; ground powders tested directly | 24- 72hrs (LC_50_, μg/ml): Extracts 9.23–53.25; powders 6.3–37.6 | Late 3rd/early 4th instar larvae of *Cx. quinquefasciatus* | _ | _ | Saponins, tannins, flavonoids, alkaloids, steroids detected qualitatively |
| [24] | 2010 | Dar es Salaa, Iringa, Mbeya, Tanga | *Ocimum suave* Willd. | Leaves, flowering | Laboratory (larvicidal, shrimp assays) | Essential oils by hydro-distillation | 24hrs (LC_50_, μg/ml) (*Cx. quinquefasciatus*): *O. suave* 151–170, *O. basilicum* 269, *O. kilimandscharicum* 323, *O. lamiifolium* 229, *A. afra* 457 | Late 3rd instar larvae of *Cx. quinquefasciatus* | _ | _ | Methyleugenol (*O. suave a*), germacrene B & D (*O. suave* b), others from the literature |
|  |  |  | *Ocimum basilicum* L. | tops, stems, bark (varied by species) |  |  |  |  |  |  |  |
|  |  |  | *Ocimum kilimandscharicum* Gürke |  |  |  |  |  |  |  |  |
|  |  |  | *Ocimum lamiifolium* Hochst. ex Benth. |  |  |  |  |  |  |  |  |
|  |  |  | *Artemisia afra* Jacq. ex Willd. |  |  |  |  |  |  |  |  |
|  |  |  | Others (11 species total) |  |  |  |  |  |  |  |  |
| [25] | 2010 | Arusha Region | *Ocimum kilimandscharicum* Gürke | Leaves | Laboratory (oviposition deterrence assays) | Essential oils by steam distillation | Tested concentrations: 2, 12, 100, 500, 1000 ppm; Oviposition activity index (OAI) ranged −0.19 to −1 | Gravid adult females, *An. gambiae* s.s. | Strong oviposition deterrence | _ | *O. kilimandscharicum* : camphor, (1,8-cineole), limonene, caryophyllene, linalool; *O. suave* : eugenol, ocimene, β-cubebene, caryophyllene, linalool (From the literature |
|  |  |  | *Ocimum suave* Willd. |  |  |  |  |  |  |  |  |
| **Study** | **Year** | **Region collected** | **Plant species** | **Plant part used** | **Study type** | **Extraction method & type** | **Concentration dose & Larval LC50** | **Mosquito species & stages** | **Repellency %** | **Adult Mortality rate** | **Phytochemicals identified** |
| [26] | 2009 | Tanga | *Uvariodendron pycnophyllum* (Diels) R.E.Fr | Stem and root barks | Laboratory (WHO larvicidal, repellency, tarsal contact) | Sequential extraction with CHCl₃ & MeOH; bioassay-guid  ed isolation- Compounds | 24-72hrs (LC_50_, μg/ml): 17–59 (*An. gambiae* larvae); extracts and compounds tested separately  Killing conc.; adults up to 200 mg/M^2^ _and_ Repellency to 10^-1^ mg/ml | Late 3rd/early 4th instar larvae & adult  *An. gambiae* | ̴ 100% | ̴ 50% | O-methyleugenol, O-methylisoeugenol, (2,3-dimethoxycinnamaldehyde), stigmasterol |
| [27] | 2006 | Pwani  Dar es salaam | *Tessmannia martiniana* Harms | Root and stem barks | Laboratory (WHO larvicidal, antimicrobial, repellency) | Sequential extraction with CHCl₃ & MeOH; bioassay-guided isolation- Compounds | LC50s (μg/ml ): Compound 3 = 1 (72h); Compound 2 = 11 (72h); Compound 5 = 256 (72h); Crude extracts = 114–256 | Late 3rd/early 4th instar larvae of *An. gambiae* s.s. | _ | _ | trans-kolavenolic acid, 18-oxocleroda-3,13(E)-dien-15-oic acid, ent-(18-hydroxycarbonyl)-cleroda-3,13(E)-dien-15-oate, 2-oxo-ent-cleroda-3,13(Z)-dien-15-oic acid, trans-2-oxo-ent-cleroda-13(Z)-en-15-oic acid, chlorobenzenoid |
| [28] | 2010 | Pwani | *Suregada zanzibariensis* Baill. | Leaves | Laboratory (human landing assays) | Essential oil by hydro-distillation | RC_50_s (×10⁻⁴ mg/cm²): verbenone 15.6, artemisia ketone 29.8, geranyl acetone 49.0; blend RC50 better than oil; essential oil RC50: 88.7 | Adult female *An. gambiae* s.s. | ̴ 90% | _ | Phenylacetaldehyde, artemisia ketone, (1S)-(-)-verbenone, geranyl acetone; total ~34 compounds identified |
| **Study** | **Year** | **Region collected** | **Plant species** | **Plant part used** | **Study type** | **Extraction method & type** | **Concentration dose & Larval LC50** | **Mosquito species & stages** | **Repellency %** | **Adult Mortality rate** | **Phytochemicals identified** |
| [29] | 2010 | Mwanza, Dar es Salaam | *Harrisonia abyssinica* Oliv. | Root and stem barks | Laboratory (WHO larvicidal & brine shrimp | DCM & EtOH extracts, bioassay-guided fractionation- Crude | 24 hrs (LC_50_, μg/ml): Not determined | Late  3rd instar larvae of  *Cx. quinquefasciatus* | _ | _ | Harrissonin, pedonin |
| [30] | 2009 | Dar es Salaam | *Annona muricata* L. | Leaves | Laboratory (WHO larvicidal & brine shrimp assays) | EtOH extraction; bioassay-guided VLC fractionation – Crude | 24hrs (LC_50_, μg/ml ) (*Cx. quinquefasciatus)*: *A. muricata* 56.47 ; *A. senegalensis* 23.42; *A. squamosa* 11.01 | Late 3rd instar larvae of *Cx quinquefasciatus* | _ | _ | Alkaloids: (-)-roemerine (*A. senegalensis*), annonaine (*A. squamosa*) |
|  |  |  | *Annona senegalensis* Pers. |  |  |  |  |  |  |  |  |
|  |  |  | *Annona squamosa* L. |  |  |  |  |  |  |  |  |
| [31] | 2009 | Pwani | *Tessmannia densiflora* Harms | Stem and root barks | Laboratory (WHO larvicidal, repellency, antimicrobial) | Sequential extraction (CHCl₃ & MeOH); bioassay-guided isolation - Compounds | (LC_50_, μg/ml ) : Tessmannic acid 93 (24h), 34 (48h); methyltessmannoate 244 (24h), 92 (48h), Adult Tessmannic acid RC50: 1×10⁻⁷ mg/cm²; methyltessmannoate RC50: 2.7×10⁻⁵ mg/cm² | Late 3rd/early 4th instar larvae & adult  *An. gambiae* | High efficacy | Tessmannic acid: 100% at 48h | Tessmannic acid, methyltessmannoate, isocoumarins (8-hydroxy-6-methoxy-3-pentylisocoumarin, 7-chloro-8-hydroxy-6-methoxy-3-pentylisocoumarin), 5-pentyl-3-methoxy-N-butylaniline |
| [32] | 2008 | Tanga, Pwani  Dar es Salaam | *Annona squamosa* L. | Stem & root barks, leaves | Laboratory (WHO larvicidal, IGR assays) | Sequential extraction (PE, CHCl₃, MeOH); bioassay-guided isolation – Compounds and Crude | 24-72hrs (LC_50_, μg/ml )varied: most active extracts from *Annona* & *Uvaria* spp. 10–100 ; e.g. *A. squamosa* RB: 13–21 | Late 3rd/early 4th instar larvae of *An. gambiae*  s.s. | _ | _ | Cbenzyl dihydrochalcones and flavanones; chamanetin, dichamanetin, melodorinol, acetylmelodorinol (*Uvaria*), 2',3'-epoxyasteranthine, 2',3'-dihydroxyasteranthine (*A. lutea*  ) prenylated indoles, Kaur-16-en-19-oic acid |
|  |  |  | *Uvaria faulknerae* Verdc. |  |  |  |  |  |  |  |  |
|  |  |  | *Uvaria kirkii* Oliv. |  |  |  |  |  |  |  |  |
|  |  |  | *Uvaria pycnophyllum* (Diels) R.E.Fr |  |  |  |  |  |  |  |  |
|  |  |  | *Tessmannia densiflora* Harms |  |  |  |  |  |  |  |  |
|  |  |  | *Asteranthe lutea*  Vollesen |  |  |  |  |  |  |  |  |
|  |  |  | The other 12 plants |  |  |  |  |  |  |  |  |
| **Study** | **Year** | **Region collected** | **Plant species** | **Plant part used** | **Study type** | **Extraction method & type** | **Concentration dose & Larval LC50** | **Mosquito species & stages** | **Repellency %** | **Adult Mortality rate** | **Phytochemicals identified** |
| [33] | 2008 | Iringa | *Synadenium glaucum* Pax | Roots, stem barks, leaves | Laboratory (WHO larvicidal assays) | Sequential extraction (Hex, DCM, MeOH); bioassay-guided isolation- Crude and Compounds | 24- 48hrs (LC_50_, μg/ml ): Steganoate A 5.44, steganacin 9.10, steganangin 10.0 (from *S. araliacea*); crude extracts varied 14–807 | Late  3rd/early 4th instar larvae of  *An. gambiae*  s.s. | _ | _ | Steganoate A, steganacin, steganangin (*A. araliacea*) |
|  |  |  | *Lantana viburnoides* (Forssk.) Vahl |  |  |  |  |  |  |  |  |
|  |  |  | *Steganotaenia araliacea* Hochst. |  |  |  |  |  |  |  |  |
|  |  |  | *Kotschya uguenensis* Verdc. |  |  |  |  |  |  |  |  |
| [34] | 2006 | Kilimanjaro | *Ocimum suave* Willd. | Leaves (dried & burned), essential oils | Field (community houses), experimental huts, lab feeding assays | Essential oils by steam distillation; burning of dried leaves | Essential oils: 20% *O. suave* or *O. kilimandscharicum* applied on skin; burning ~1 kg dried leaves per night | Adults of *An. arabiensis*, *Cx. quinquefasciatus* | *An. arabiensis*: *O. suave* 91.98%, *O. kilimandscharicum,* 89.75%; *vs Cx. quinquefasciatus*: *O. suave* 88.65%, *O. kilimandscharicum* 90.50%. | _ | *O. suave*: linalool-rich; *O. kilimandscharicum* : camphor-rich (literature) |
|  |  |  | *Ocimum kilimandscharicum* Gürke |  |  |  |  |  |  |  |  |
|  |  |  | *Azadirachta indica* A.Juss. |  |  |  |  |  |  |  |  |
|  |  |  | *Eucalyptus globulus* Labill. |  |  |  |  |  |  |  |  |
|  |  |  | *Lantana camara* L. |  |  |  |  |  |  |  |  |
| [35] | 2006 | Kilimanjaro | *Ocimum suave* Willd. | Leaves (fresh, burned, essential oils) | Ethnobotanical survey, lab cone & tunnel assays, cage tests | Steam distillation; netting & filter paper impregnation | Dosages: 75–500 mg/m²; feeding inhibition *O. suave*: 83.5–88.9%, *O. kilimandscharicum* : 71.2–85.3%; KD_90_ *O. suave* : ~14–21 min, *O. kilimandscharicum* : ~15–20 min; | Female adults of *An. gambiae* s.s., *An. arabiensis*, *Cx. quinquefasciatus* | *O. suave* : 83–91%; *O. kilimandscharicum* : 71–92% repellency depending on species | 47.3 - 65% for *O. kilimandscharicum*  and 50-67% for *O. suave* | *O. suave* : eugenol, linalool; *O. kilimandscharicum* : camphor, 1,8-cineole, limonene (literature) |
|  |  |  | *Ocimum kilimandscharicum* Gürke |  |  |  |  |  |  |  |  |
| **Study** | **Year** | **Region collected** | **Plant species** | **Plant part used** | **Study type** | **Extraction method & type** | **Concentration dose & Larval LC50** | **Mosquito species & stages** | **Repellency %** | **Adult Mortality rate** | **Phytochemicals identified** |
| [36] | 2008 | Iringa | *Lantana viburnoides* var kisi (A.Rich.) Verdc | Root barks | Laboratory (WHO larvicidal assays) | Sequential extraction (Hex, DCM, MeOH); VLC fractions- Crude and Compounds | (LC_50_, μg/ml ): (72h): crude extract 7.7; active fractions: furanonaphthaquinones 5.48–5.7, camaric acid 6.19, betulinic acid <10 | Late 3rd/early 4th instar larvae of *An. gambiae*  s.s. | _ | _ | Furanonaphthaquinones (regio-isomers), camaric acid, betulinic acid |
| [37] | 2004 | Pwani | *Hugonia busseana* Gilg. | Root barks | Laboratory (brine shrimp & WHO larvicidal bioassays) | EtOH extraction; VLC, DCM fractionation, Sephadex LH-20, Compounds | LC50s (μg/ml ):  ; moderate activity at 237 (24h)  Complete mortality at 13.69 mg/mL (hugonianene A) | 3rd/4th instar larvae of *An. gambiae* | _ | _ | Hugonianene A (himachalene-type), 18-hydroxyrosane, hugorosediol, hugonone B, podocarpane derivatives |
| [38] | 2005 | Morogoro | *Tephrosia vogelii* Hook.f. | Leaf | Larvicidal bioassay | Water extract (distilled & sep- tic tank water)- Crude | 10% in EtOH | 3rd stage larvae *Cx. quinquefasciatus*, | _ | _ | _ |
| [39] | 2004 | Tanga | *Ocimum suave* Willd. | Leaves | Field & community trials | Essential oils by Steam distillation | Applied to human skin; protective efficacy lasted 6–8 hours; dose not specified | Adult female *An. gambiae* s.s., *An*. *funestus, Cx. quinquefasciatus* | 83% in *An. gambiae*, 100% in *An. funestus*) and 75% in *Cx. quinquefasciatus* | _ | Linalool, 1,8-cineole, eugenol, other monoterpenes (from literature) |
| [40] | 2004 | Tanga | *Uvaria scheffleri* Diels | Root bark | Laboratory(WHO larvicidal bioassays) | PE extract; VLC- Compounds | LC50 (µg/mL) 930.5 (48h), 18.3 (72h), 0.5 (96h) | *An. gambiae* (late 3rd/early 4th instar larvae) | _ | _ | (±)-Schefflone, espintanol |
| [41] | 2004 | Iringa | *Neorautanenia mitis* (A.Rich.) Verdc. | Tuber | Laboratory(WHO larvicidal bioassays) | PE, DCM, EtOH; VLC, FC, Sephadex LH-20- Componds | LC50 (µg/mL)  *An. gambiae* = 68 (PE), 110 (DCM); *Cx. quinquefasciatus* = 160 (PE), 260 (DCM) | *An. gambiae & Cx. quinquefasciatus* (larvae), *An. gambiae* (adult) | _ | ̴ 100% | Pachyrrhizine, neotenone, neorautanone, neoduline, 4-methoxyneoduline, nepseudin |

**References**

[1] B. Emidi, Efficacy of *Chenopodium ambrosioides* essential oil against *Anopheles gambiae* sensu lato, *Anopheles funestus*, and *Aedes aegypti* mosquitoes in Muheza, Tanzania, Tanzan. J. Health Res. 26 (2025) 2017–2122. https://www.ajol.info/index.php/thrb/article/view/285619 (accessed July 3, 2025).

[2] A.G. Sanga, H.D. Mazigo, A. Manjurano, D. Morona, A. Thomas, E.J. Kweka, Measuring repellence and mortality effects of clove and cinnamon essential oils impregnated nets against *Anopheles gambiae* senso stricto using tunnel test, J. Nat. Pestic. Res. 5 (2023) 100046. https://doi.org/10.1016/j.napere.2023.100046.

[3] B. Samwel, E. Innocent, F. Machumi, W.N. Kisinza, M. Heydenreich, Two mosquito larvicidal arabinofuranosidetridecanol from *Commiphora merkeri* exudate, Nat. Prod. Res. 36 (2022) 2821–2829. https://doi.org/10.1080/14786419.2021.1931866.

[4] A. Philbert, Larvicidal potency of *Dioscorea sansibarensis* leaf extract against vector mosquitoes: *Anopheles gambiae* s.s. and C*ulex quinquefasciatus*, Tanzan. J. Sci. 47 (2021) 655–663. https://doi.org/10.4314/tjs.v47i2.21.

[5] I.J. Daniel, E. Innocent, J. Sempombe, V. Mugoyela, T. Fossen, Isolation and characterization of larvicidal phenolic acids from *Kotschya thymodora* leaves, J. Appl. Sci. Environ. Manag. 24 (2020) 1483–1488. https://doi.org/10.4314/jasem.v24i8.26.

[6] A.J. Sillo, W.E. Makirita, H. Swai, M. Chacha, Larvicidal activity of *Hypoestes forskaolii* (Vahl) R. Br root extracts against *Anopheles gambiae* Giless.s, *Aedes* aegypti L, and *Culex quinquefasciatus* Say, J. Exp. Pharmacol. (2019). https://www.tandfonline.com/doi/abs/10.2147/JEP.S187837 (accessed July 3, 2025).

[7] B. Samwel, E. Innocent, F. Machumi, W.N. Kisinza, Mosquito larvicidal and brine shrimp activities of *Commiphora merkeri* Engl. (Burseraceae) exudate, Int. J. Mosq. Res. 6 (2019) 01–04. https://www.dipterajournal.com/archives/2019/6/2/A/6-1-14 (accessed July 6, 2025).

[8] E.A. Inocente, B. Nguyen, P.K. Manwill, A. Benatrehina, E. Kweka, S. Wu, X. Cheng, L.H. Rakotondraibe, P.M. Piermarini, Insecticidal and antifeedant activities of Malagasy medicinal plant (*Cinnamosma* sp.) extracts and drimane-type sesquiterpenes against *Aedes aegypti* mosquitoes, Insects 10 (2019) 373. https://doi.org/10.3390/insects10110373.

[9] A. Thomas, H.D. Mazigo, A. Manjurano, D. Morona, E.J. Kweka, Evaluation of active ingredients and larvicidal activity of clove and cinnamon essential oils against *Anopheles gambiae* (sensu lato), Parasit. Vectors 10 (2017) 411. https://doi.org/10.1186/s13071-017-2355-6.

[10] M. Mkangara, M. Chacha, P. Kazyoba, Larvicidal potential of *Commiphora swynnertonii* (Burtt) Stem Bark Extracts against *Anopheles gambiae* ss, *Culex quinquefasciatus* Say and *Aedes aegypti* L, Int. J. Sci. Res. IJSR volume 4 (2015) 356–361.

[11] W. Leonidas, J. Shipili, J. Saria, Y. Lawi, E. Kweka, F. Magogo, W. Kisinza, H. Malebo, Bioprospection for repellent effect of natural volatiles from *Ocimum suave* Willd growing in Dar es Salaam, Tanzania against *Anopheles* mosquitoes, Int. J. Trop. Dis. Health 6 (2015) 73–79. https://doi.org/10.9734/IJTDH/2015/14710.

[12] A. Kidukuli, S. Maregesi, J. Saria, J. Otieno, L. Yohana, R. Nondo, E. Innocent, J. Mlimbila, M. Mihale, M. Moshi, Larvicidal efficacy of some *Tehrosia* species extracts against *Anopheles gambiae* ss and *Culex quinquefasciatus* say, Spatula DD 5 (2015) 21–25. https://doi.org/10.5455/spatula.20150625013601.

[13] E. Innocent, A. Hassanali, Constituents of essential oils from three plant species used in traditional medicine and insect control in Tanzania, J. Herbs Spices Med. Plants 21 (2015) 219–229. https://doi.org/10.1080/10496475.2014.949997.

[14] R.A. Suleiman, Q.A. Mgani, S.S. Nyandoro, Chemical compositions and mosquito repellency of essential oils from *Artabotrys hexapetalus* and *Artabotrys rupestris*, Int. J. Biol. Chem. Sci. 8 (2014) 2804–2812. https://doi.org/10.4314/ijbcs.v8i6.37.

[15] J.W. Nkya, P. Erasto, M. Chacha, Larvicidal against mosquito vectors and brine shrimp activities of extracts from the flowers of *Moringa oleifera* Lam, Am. J. Res. Commun. 2 (2014).

[16] E. Innocent, A. Hassanali, W.N. Kisinza, P.P. Mutalemwa, S. Magesa, E. Kayombo, Anti-mosquito plants as an alternative or incremental method for malaria vector control among rural communities of Bagamoyo District, Tanzania, J. Ethnobiol. Ethnomedicine 10 (2014) 56. https://doi.org/10.1186/1746-4269-10-56.

[17] S.S. Nyandoro, C.C. Joseph, M.H.H. Nkunya, K.M.M. Hosea, New antimicrobial, mosquito larvicidal and other metabolites from two *Artabotrys* species, Nat. Prod. Res. 27 (2013) 1450–1458. https://doi.org/10.1080/14786419.2012.725397.

[18] H.M. Malebo, C. Imeda, N.A. Kitufe, S.J. Katani, R. Sunguruma, F. Magogo, P.K. Tungu, V.A. Nyigo, V. Wiketye, G.L. Mwaiko, J.W. Ogondiek, G.P. Mbogo, P.P. Mhame, D.Z. Matata, R. Malima, S.M. Magesa, J.J. Massaga, M.N. Malecela, rew Y. Kitua, Repellence effectiveness of essential oils from some Tanzanian *Ocimum* and *Hyptis* plant species against afro-tropical vectors of malaria and lymphatic filariasis, J. Med. Plants Res. 7 (2013) 653–660. https://doi.org/10.5897/JMPR11.732.

[19] E.J. Kweka, S. Munga, A.M. Mahande, S. Msangi, H.D. Mazigo, A.Q. Adrias, J.R. Matias, Protective efficacy of menthol propylene glycol carbonate compared to N, N-diethyl-methylbenzamide against mosquito bites in Northern Tanzania, Parasit. Vectors 5 (2012) 189. https://doi.org/10.1186/1756-3305-5-189.

[20] R.S.O. Nondo, Z.H. Mbwambo, A.W. Kidukuli, E.M. Innocent, M.J. Mihale, P. Erasto, M.J. Moshi, Larvicidal, antimicrobial and brine shrimp activities of extracts from *Cissampelos mucronata* and *Tephrosia villosa* from coast region, Tanzania, BMC Complement. Altern. Med. 11 (2011) 33. https://doi.org/10.1186/1472-6882-11-33.

[21] F.C. Mng’ong’o, J.J. Sambali, E. Sabas, J. Rubanga, J. Magoma, A.J. Ntamatungiro, E.L. Turner, D. Nyogea, J.H.J. Ensink, S.J. Moore, Repellent plants provide affordable natural screening to prevent mosquito house entry in tropical rural settings—results from a pilot efficacy study, PLoS ONE 6 (2011) e25927. https://doi.org/10.1371/journal.pone.0025927.

[22] C. Kihampa, Tanzanian botanical derivatives in the control of malaria vectors: opportunities and challenges, J. Appl. Sci. Environ. Manag. 15 (2011). https://doi.org/10.4314/jasem.v15i1.65694.

[23] B. Daniel, E. Innocent, Z.H.M.A.S.G. Musharraf, Comparison of mosquito larvicidal activity of *Annona squamosa* leaves growing in different eco-zones in Tanzania, Int. J. Pharma Bio Sci. Volume 2 Issue 4 (2011). http://www.ijpbs.net/abstract.php?article=1108 (accessed July 4, 2025).

[24] D.K.B. Runyoro, O. Ngassapa, L. Kachali, V. Obare, E.F. Lyamuya, Biological activities of essential oils from plants growing in Tanzania, East Cent. Afr. J. Pharm. Sci. 13 (2010). https://www.ajol.info/index.php/ecajps/article/view/107359 (accessed July 3, 2025).

[25] E.J. Kweka, E. Lyatuu, M. Mboya, B. Mwang′onde, A. Mahande, Oviposition deterrence induced by *Ocimum kilimandscharicum* and O*cimum suave* extracts to gravid *Anopheles gambiae* s.s (Diptera: Culicidae) in laboratory, J. Glob. Infect. Dis. 2 (2010) 242. https://doi.org/10.4103/0974-777X.68524.

[26] C. Kihampa, M.H.H. Nkunya, C. Joseph, S. Magesa, Antimosquito phenylpropenoids from the stem and root barks of *Uvariodendron pycnophyllum* (Diels) R.E.Fr, J. Appl. Sci. Environ. Manag. 14 (2010). https://doi.org/10.4314/jasem.v14i3.61457.

[27] C. Kihampa, M.H.H. Nkunya, C.C. Joseph, S.M. Magesa, A. Hassanali, M. Heydenreiche, E. Kleinpeter, Antimosquito and antimicrobial clerodanoids and a chlorobenzenoid from Tessmannia species, Nat. Prod. Commun. 5 (2010) 175–178.

[28] E. Innocent, C.C. Joseph, N.K. Gikonyo, M.H.H. Nkunya, A. Hassanali, Constituents of the essential oil of *Suregada zanzibariensis* leaves are repellent to the mosquito, *Anopheles gambiae* s.s., J. Insect Sci. 10 (2010) 57. https://doi.org/10.1673/031.010.5701.

[29] E. Innocent, J.J. Magadula, Variations of anti-mosquito larvicidal constituents in the *Harrisonia abyssinica* species of Tanzania, Int. J. Biol. Chem. Sci. 4 (2010). https://doi.org/10.4314/ijbcs.v4i1.54249.

[30] J.J. Magadula, Mosquito larvicidal and cytotoxic activities of 3 *Annona* species and isolation of active principles, J. Med. Plants Res. 3 (2009) 674–680. http://www.academicjournals.org/JMPR.

[31] C. Kihampa, M.H.H. Nkunya, C.C. Joseph, S.M. Magesa, A. Hassanali, M. Heydenreich, E. Kleinpeter, Anti-mosquito and antimicrobial nor-halimanoids, isocoumarins and an anilinoid from T*essmannia densiflora*, Phytochemistry 70 (2009) 1233–1238. https://doi.org/10.1016/j.phytochem.2009.07.024.

[32] C. Kihampa, C.C. Joseph, M.H.H. Nkunya, S.M. Magesa, A. Hassanali, M. Heydenreich, E. Kleinpeter, Larvicidal and IGR activity of extract of Tanzanian plants against malaria vector mosquitoes, J VECTOR BORNE DIS (2009).

[33] E. Innocent, J.C. Cosam, N.K. Gikonyo, M.H. Nkunya, A. Hassanali, Larvicidal properties of some Tanzanian plant species against *Anopheles gambiae* s.s. Gile (Diptera: Culicidae) mosquitoes, Int. J. Biol. Chem. Sci. 3 (2009). https://doi.org/10.4314/ijbcs.v3i2.44510.

[34] E.J. Kweka, F.W. Mosha, A. Lowassa, A.M. Mahande, M.J. Mahande, C.P. Massenga, F. Tenu, E.E. Lyatuu, M.A. Mboya, E.A. Temu, Longitudinal evaluation of *Ocimum* and other plants effects on the feeding behavioral response of mosquitoes (Diptera: Culicidae) in the field in Tanzania, Parasit. Vectors 1 (2008) 42. https://doi.org/10.1186/1756-3305-1-42.

[35] E.J. Kweka, F. Mosha, A. Lowassa, A.M. Mahande, J. Kitau, J. Matowo, M.J. Mahande, C.P. Massenga, F. Tenu, E. Feston, E.E. Lyatuu, M.A. Mboya, R. Mndeme, G. Chuwa, E.A. Temu, Ethnobotanical study of some of mosquito repellent plants in north-eastern Tanzania, Malar. J. 7 (2008) 152. https://doi.org/10.1186/1475-2875-7-152.

[36] E. Innocent, C.C. Joseph, N.K. Gikonyo, M.J. Moshi, M.H.H. Nkunya, A. Hassanali, Mosquito larvicidal constituents from *Lantana viburnoides sp viburnoides* var kisi (A. rich) Verdc (Verbenaceae), J. Vector Borne Dis. 45 (2008) 240–244.

[37] L.D. Baraza, C.C. Joseph, M.H.H. Nkunya, A new cytotoxic and larvicidal himachalenoid, rosanoids and other constituents of *Hugonia busseana*, Nat. Prod. Res. 21 (2007) 1027–1031. https://doi.org/10.1080/14786410701371074.

[38] B. Kabula, B.S. Kilonzo, Potential larvicidal effects of tephrosia vogelii leaf extract on *Culex quinquefasciatus* in Morogoro, Tanzania, Tanzan. J. Health Res. 7 (2005). https://doi.org/10.4314/thrb.v7i1.45103.

[39] H. Malebo, Nguruwe, Lugimbana, Sambu, M. Malecela, M. Kamugisha, Senkoro, Magesa, Efficacy of *Ocimum suave* volatile oil formulation against man-biting mosquitoes in Muheza, north-east Tanzania, Tanzan. Health Res. Bull. 7 (2005) 25. https://doi.org/10.4314/thrb.v7i1.45104.

[40] M.H.H. Nkunya, S.A. Jonker, R. De Gelder, S.W. Wachira, C. Kihampa, (±)-Schefflone: a trimeric monoterpenoid from the root bark of *Uvaria scheffleri*, Phytochemistry 65 (2004) 399–404. https://doi.org/10.1016/j.phytochem.2003.10.011.

[41] C.C. Joseph, M.M. Ndoile, R.C. Malima, M.H.H. Nkunya, Larvicidal and mosquitocidal extracts, a coumarin, isoflavonoids and pterocarpans from *Neorautanenia mitis*, Trans. R. Soc. Trop. Med. Hyg. 98 (2004) 451–455. https://doi.org/10.1016/j.trstmh.2003.10.008.
